# *Chamaecyparis lawsoniana* and Its Active Compound Quercetin as Ca^2+^ Inhibitors in the Contraction of Airway Smooth Muscle

**DOI:** 10.3390/molecules29102284

**Published:** 2024-05-12

**Authors:** Edgar Flores-Soto, Bianca S. Romero-Martínez, Héctor Solís-Chagoyán, Edgar A. Estrella-Parra, Jose G. Avila-Acevedo, Juan C. Gomez-Verjan, Jorge Reyes-García, María F. Casas-Hernández, Bettina Sommer, Luis M. Montaño

**Affiliations:** 1 Departamento de Farmacología, Facultad de Medicina, Universidad Nacional Autónoma de México, Avenida Universidad No. 3000, Alcaldía de Coyoacán, C.P., Mexico City 04510, Mexico; edgarfloressoto@yahoo.com.mx (E.F.-S.); biancasromero_@hotmail.com (B.S.R.-M.); reyes.garcia.jorge@gmail.com (J.R.-G.); fercasashdz@hotmail.com (M.F.C.-H.); 2 Centro de Investigación en Ciencias Cognitivas, Universidad Autónoma del Estado de Morelos, Avenida Universidad 1001, Col. Chamilpa, C.P., Cuernavaca 62209, Mexico; hecsolch@imp.edu.mx; 3 Laboratorio de Fitoquímica, Unidad de Biología Tecnología y Prototipos, FES-Iztacala, Universidad Nacional Autónoma de México, Avenida de los Barrios No. 1, Los Reyes Iztacala, C.P., Tlalnepantla 54090, Mexico; estreparr@iztacala.unam.mx (E.A.E.-P.); tuncomaclovio2000@yahoo.com.mx (J.G.A.-A.); 4 Dirección de Investigación, Instituto Nacional de Geriatría (INGER), Anillo Periférico. 2767, San Jerónimo Lídice, La Magdalena, C.P., Mexico City 10200, Mexico; jverjan@inger.gob.mx; 5 Departamento de Investigación en Hiperreactividad Bronquial, Instituto Nacional de Enfermedades Respiratorias “Ismael Cosio Villegas”, Calz. De Tlalpan 4502, Col. Sección XVI, Alcaldía de Tlalpan, C.P., Mexico City 14080, Mexico

**Keywords:** *Chamaecyparis lawsoniana*, quercetin, airway smooth muscle relaxation, store-operated Ca^2+^ channels, L-type voltage-dependent Ca^2+^ channels

## Abstract

The *Cupressaceae* family includes species considered to be medicinal. Their essential oil is used for headaches, colds, cough, and bronchitis. Cedar trees like *Chamaecyparis lawsoniana* (*C. lawsoniana*) are commonly found in urban areas. We investigated whether *C. lawsoniana* exerts some of its effects by modifying airway smooth muscle (ASM) contractility. The leaves of *C. lawsoniana* (363 g) were pulverized mechanically, and extracts were obtained by successive maceration 1:10 (*w*:*w*) with methanol/CHCl_3_. Guinea pig tracheal rings were contracted with KCl, tetraethylammonium (TEA), histamine (HIS), or carbachol (Cch) in organ baths. In the Cch experiments, tissues were pre-incubated with D-600, an antagonist of L-type voltage-dependent Ca^2+^ channels (L-VDCC) before the addition of *C. lawsoniana*. Interestingly, at different concentrations, *C. lawsoniana* diminished the tracheal contractions induced by KCl, TEA, HIS, and Cch. In ASM cells, *C. lawsoniana* significantly diminished L-type Ca^2+^ currents. ASM cells stimulated with Cch produced a transient Ca^2+^ peak followed by a sustained plateau maintained by L-VDCC and store-operated Ca^2+^ channels (SOCC). *C. lawsoniana* almost abolished this last response. These results show that *C. lawsoniana*, and its active metabolite quercetin, relax the ASM by inhibiting the L-VDCC and SOCC; further studies must be performed to obtain the complete set of metabolites of the extract and study at length their pharmacological properties.

## 1. Introduction

Studies regarding phytochemical agents popularly used among the general public have gained interest in the scientific community in recent decades. Traditional medicinal practices have ancestral origins and open new venues for effective and affordable treatment options [1,2]. *Chamaecyparis lawsoniana* (*C. lawsoniana* (A. Murray bis) Parl.), a member of the *Cupressaceae* family, is a perennial native to North America and eastern Asia, and the largest species is the Lawson cypress, commonly known as Port Orford cedar, or ginger pine (*Chamaecyparis lawsoniana*), reaching a height up to 60 m (200 feet) and about 2 m (70 inches) in diameter. This pine tree is native to southwestern Oregon and northern California [3].

This species was introduced to Mexico, where it is well-adapted and commercially cultivated as a Christmas tree for ornamental purposes. From the pharmacological point of view, this tree has been studied through ex vivo experiments investigating its anti-inflammatory, antiviral, and antibacterial properties. Another member of this genus, the *Chamaecyparis obtusa* (*C. obtuse* Siebold & Zucc.), has also been studied and is attributed with anti-inflammatory [4] and vascular smooth muscle anti-proliferative properties [5], pointing out their pharmacological potential. It has been established that the essential oil of *Chamaecyparis* spp. possesses antibacterial capacities [6], and an ethanolic extract of *C. lawsoniana* showed antiviral action against Herpes simplex virus type 2 [7], highlighting its importance in alternative medicine. In this sense, asthma is a respiratory disease characterized by a chronic inflammation including the activation of the Th2 cell response to allergens, and is often found to have elevated levels of inflammatory cytokines including IL-4, IL-5, IL-13, TNF-α, and IL-1β, among others [8]. Conifer species have been reported to have anti-inflammatory, antioxidant, antiproliferative, antibacterial, and antiparasitic activities. In ovalbumin-sensitized mouse asthma models, exposure to wood panels of *C. obtusa* decreased levels of IL-4, IL-9, IL-13, and TNF-α [9]. Furthermore, in lipopolysaccharide-induced inflammation in mice, *C. obtusa* volatile organic compounds diminished the levels of cyclooxygenase 2, IL-1β, IL-13, and TNF-α [9,10]. In addition to these attributes, we found that the *C. lawsoniana* methanolic extract could inhibit the contraction of the airway smooth muscle (ASM). Moreover, Port Oxford cedar heartwood oil at a concentration as high as 5 mg/L (the highest concentration tested due to its solubility limit) demonstrated no toxicity risk on *Daphnia magna*, *Oncorhynchus mykiss*, and *Selenastrum capricornatum* [11].

The contraction of ASM is coordinated by increased intracellular Ca^2+^ levels, sensitization of Ca^2+^, filament interaction, cytoskeletal remodeling, and activation of signaling pathways [12]. Several agonists such as acetylcholine, histamine, bradykinin, leukotrienes, substance P, ATP, etc. generate ASM sustained contraction through the activation of G protein-coupled receptors (GPCR)-qα, inducing an initial transient Ca^2+^ peak followed by a plateau, with the subsequent sustained contraction [13,14,15]. This cytoplasmic Ca^2+^ binds with calmodulin to form a Ca^2+^-calmodulin complex, activating the myosin light-chain kinase (MLCK). This activated MLCK phosphorylates the myosin light chain (LC20) to induce cross-bridge cycling, which results in the contraction of the smooth muscle. The sustained contraction of ASM is related to a Ca^2+^ sensitization phenomenon [16,17,18].

In guinea pig ASM, the agonist-induced Ca^2+^ plateau is mainly related to the activation of L-type voltage-dependent Ca^2+^ channels (L-VDCC) and store-operated Ca^2+^ channels (SOCC) and is, therefore, extracellular Ca^2+^ dependent. These channels have been involved in smooth muscle membrane depolarization and mediate Ca^2+^ influx to trigger a sustained contraction of the ASM [19,20,21,22]. We aimed to explore the effects of *C. lawsoniana* on tracheal smooth muscle contraction.

## 2. Results

### 2.1. C. lawsoniana Methanolic (MeOH) Extract Diminishes KCl and TEA-Induced Contractions in Guinea Pig Trachea

**Figure 1:**
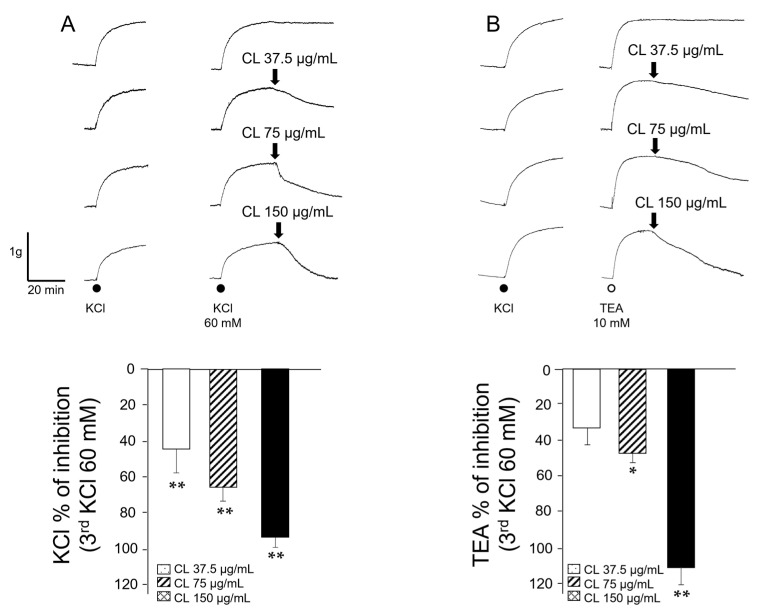
*C. lawsoniana* methanolic extract concentration-dependently diminishes KCl and tetraethylammonium (TEA)-induced contraction in guinea pig tracheal smooth muscle. (**A**) The upper panel shows original traces of the effects of different *C. lawsoniana* (CL) concentrations (37.5, 75, and 150 µg/mL) on the 60 mM KCl-induced contraction. Below, bar graph illustrating the significant diminution in KCl-induced tension produced by each CL concentration tested (*n* = 7). (**B**) Original recordings of the CL effects on the TEA (10 mM, *n* = 5–6) induced contraction. Bar graph depicts the significances reached for CL effect at 75 µg/mL and 150 µg/mL. Bars represent mean ± SEM, * *p* < 0.05; ** *p*< 0.01. Results are expressed as a percentage of the third KCl response.

Tracheas were pre-contracted with KCl or TEA (a non-selective blocker of the K^+^ channels); both substances induced membrane depolarization and, therefore, external Ca^2+^ entry and contraction through L-VDCC activation. Contractile responses induced by KCl 60 mM were significantly (*p* < 0.01) diminished by the addition of every *C. lawsoniana* concentration (37.5, 75, and 150 µg/mL) tested (Figure 1A). When tracheas were contracted with TEA, significant relaxation responses were seen for 75 µg/mL (*p* < 0.05) and 150 µg/mL of *C. lawsoniana* (*p* < 0.01) (Figure 1B).

### 2.2. C. lawsoniana Significantly Lowers Histamine-Induced Contraction in Tracheal Preparations

**Figure 2:**
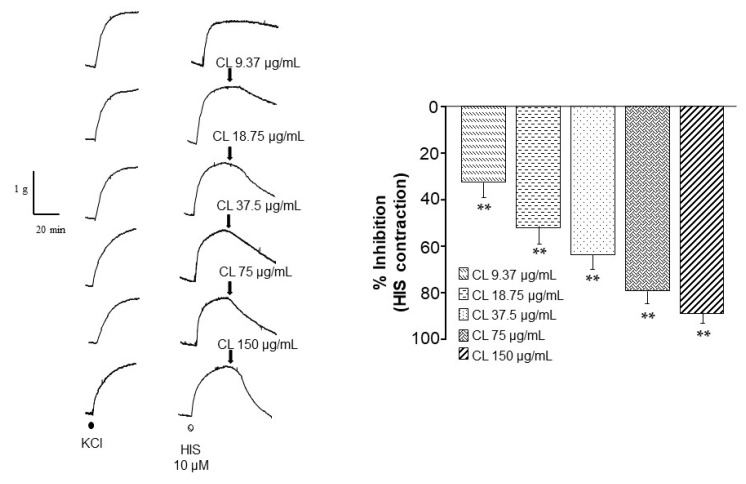
*C. lawsoniana* methanolic extract diminishes histamine-induced contractions in guinea pig airway smooth muscle. The left panel illustrates original traces of the effects of different *C. lawsoniana* (CL) concentrations (9.37, 18.75, 37.5, 75, and 150 µg/mL) on the histamine (HIS, 10 µM, *n* = 6–7) induced contraction. On the right, bar graph depicting the significance reached at all CL concentrations used. Bars represent mean ± SEM, ** *p* < 0.01.

Histamine (HIS)-induced tracheal contractions were significantly (*p* < 0.01) lowered by all *C. lawsoniana* concentrations (9.37, 18.75, 37.5, 75, and 150 µg/mL) (Figure 2).

### 2.3. Store-Operated Ca^2+^ Channels Are Blocked by C. lawsoniana Methanolic Extract 

**Figure 3:**
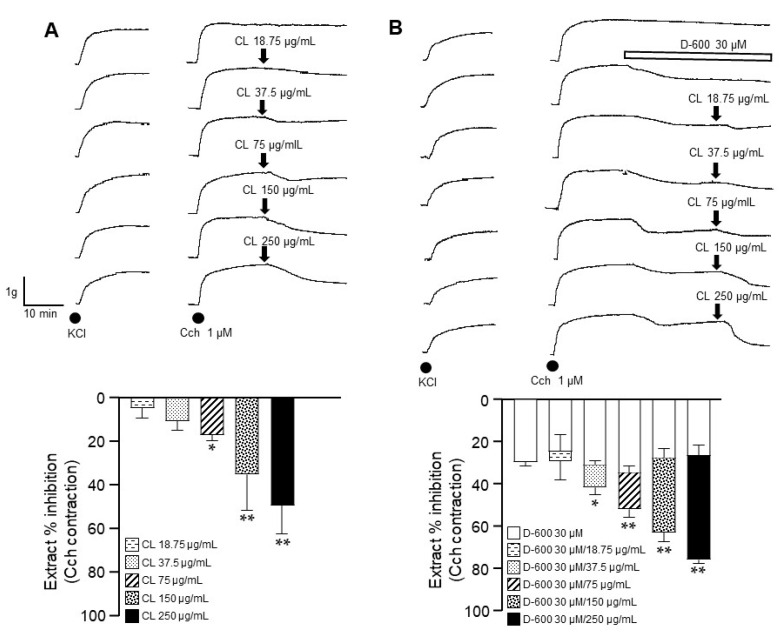
Store-operated Ca^2+^ channels (SOCC) might be blocked by the methanolic extract of *C. lawsoniana* (CL). (**A**) The upper panel shows original traces of smooth muscle contraction induced by Cch 1 µM and the effect of different CL concentrations (18.75, 37.5, 75, 150, and 250 µg/mL) (*n* = 6). The bar graph summarizes the data analysis and significant differences observed for CL 75, 150, and 250 µg/mL. (**B**) Original traces of smooth muscle contraction induced by Cch 1 µM and the relaxation developed by D-600 (30 µM) used to block L-type voltage-dependent Ca^2+^ channels (L-VDCC) and the addition of CL (18.75, 37.5, 75, 150, and 250 µg/mL) (*n* = 6). Note that D-600 caused a new plateau that was almost abolished by the different CL concentrations tested, indicating a possible inhibition of store-operated Ca^2+^ channels (SOCC). The bar graph in the right panel illustrates the relaxation induced by D-600 alone and after the addition of each CL concentration after D-600. Bars represent mean values ± SEM, * *p* < 0.05, ** *p* < 0.01.

Charbacol (Cch)-induced tracheal contractions were significantly lowered by *C. lawsoniana* (75, 150, and 250 µg/mL) (Figure 3A).

The addition of D-600 during the maximal contraction induced by Cch produced a relaxation of around 26% due to the blockade of L-VDCC. Once this relaxation reached a plateau, the administration of 9.37, 18.75, 37.5, 75, 150, or 250 µg/mL of methanolic extract of *C. lawsoniana* induced a concentration-dependent relaxation of the Cch-induced contraction. The addition of the CL extract had a significant relaxation at the highest concentrations tested (37.5, 75, 150, and 250 µg/mL) (Figure 3B). These last results suggest that the relaxation induced by *C. lawsoniana* could be due to the blockade of the store-operated Ca^2+^ channels (SOCC). 

### 2.4. C. lawsoniana Blocks L-Type Ca^2+^ Currents but Not K^+^ Currents in Tracheal Myocytes

**Figure 4:**
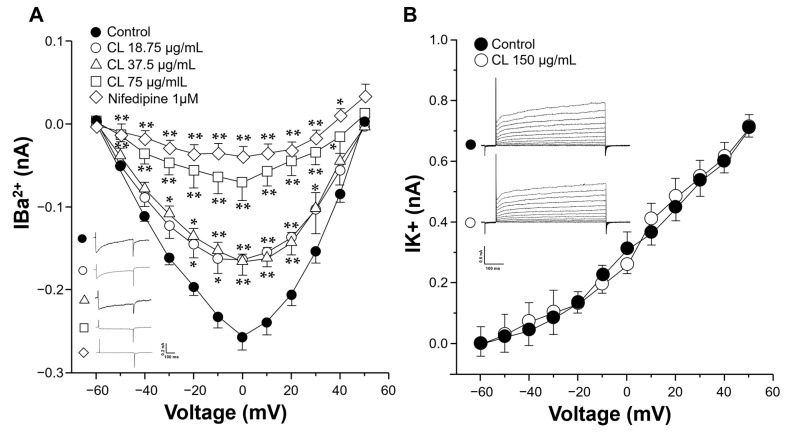
Voltage-dependent L-type Ca^2+^ currents, but not K^+^ currents, were diminished by the *C. lawsoniana* (CL) methanolic extract in tracheal myocytes. (**A**) Cultured guinea pig tracheal myocytes were held at a membrane potential of − 60 mV, and then depolarizing pulses were applied in 10 mV steps to +50 mV. This caused voltage-dependent Ba^2+^ inward currents (IBa^2+^) corresponding to L-type Ca^2+^ currents, as 1 µM nifedipine abolished them. The peak inward current reached its maximum amplitude at 0 mV. When multiple concentrations of CL extract (*n* = 5–7) were perfused, these currents decreased, reaching significance compared to the control current. The original recordings for each concentration tested are shown as insets. The symbols indicate the mean ± SEM, where * *p* < 0.05 and ** *p* < 0.01 compared to the control group. (**B**) The cultured cells received a step depolarization protocol from −60 to +50 mV in 10 mV increments from a holding potential of −60 mV during 500 ms. These stimulations generated a voltage-dependent outward K^+^ current (IK^+^). Myocytes perfused with the methanolic extract of *C. lawsoniana* (CL, 150 μg/mL) showed no changes in IK^+^ compared to the control group (*n* = 3). The insets in the figure show the original traces of IK^+^.

In the voltage clamp experiments with tracheal myocytes, step depolarizations from −60 to +50 mV from a holding potential of −60 mV induced a voltage-dependent inward Ba^2+^ current (IBa^2+^). This current corresponded to L-VDCC activity as the addition of 1 µM nifedipine (a blocker of L-VDCC) completely annulled this response. The peak inward current reached its maximum amplitude at 0 mV. When tracheal myocytes were exposed to 18.75, 37.5, or 75 µg/mL of *C. lawsoniana* extract, a significant reduction in each current was seen until its virtual abolition with the higher concentration (Figure 4A). Additionally, outward K^+^ currents were generated by depolarization pulses (from −60 to +50 mV) in tracheal myocytes. However, when the myocytes were perfused with the *C. lawsoniana* extract (CL, 150 μg/mL), these K^+^ currents were not affected (Figure 4B).

### 2.5. C. lawsoniana Blocks L-Type Voltage-Dependent Ca^2+^ Channels and Store-Operated Ca^2+^ Channels Diminishing Intracellular Ca^2+^ Concentration in Tracheal Myocytes

**Figure 5:**
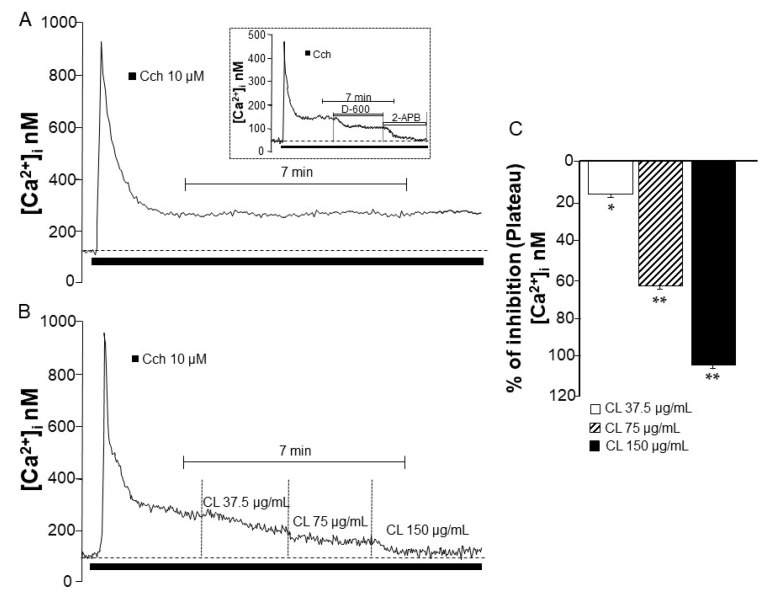
Both L-type voltage-dependent Ca^2+^ channels (L-VDCC) and store-operated Ca^2+^ channels (SOCC) were blocked by the methanolic extract of *C. lawsoniana* (CL) in guinea pig tracheal myocytes. (**A**) Once stimulated with carbachol (Cch), myocytes show an intracellular Ca^2+^ peak followed by a plateau. The inset shows that this Ca^2+^ plateau is sustained by L-VDCC, blocked by D-600 (30 µM), and SOCC, blocked by 2-APB (100 µM). (**B**) Cumulative addition of CL concentrations lowered the Cch-induced Ca^2+^ plateau (*n* = 5), indicating that this extract blocks both L-VDCC and SOCC in tracheal myocytes. (**C**) Bar graph illustrating CL effects on the Cch-induced Ca^2+^ plateau. Bars represent mean ± SEM, * *p*< 0.05, ** *p* < 0.01.

Single tracheal myocytes stimulated with Cch (10 µM) produced a transient Ca^2+^ peak followed by a plateau (Figure 5A). It is well-known that this Ca^2+^ plateau is related to sustained contraction induced by different agonists including Cch [15]. This Cch-induced plateau was abolished when D-600 and 2-APB were added (inset Figure 5), demonstrating the participation of L-VDCC and SOCC in this response. The cumulative addition of *C. lawsoniana* concentrations significantly lowered the Cch-induced Ca^2+^ plateau (Figure 5C), indicating that this extract blocks both L-VDCC and SOCC in tracheal myocytes. 

### 2.6. Phytochemical Composition of C. lawsoniana Methanolic Extract

**Figure 6:**
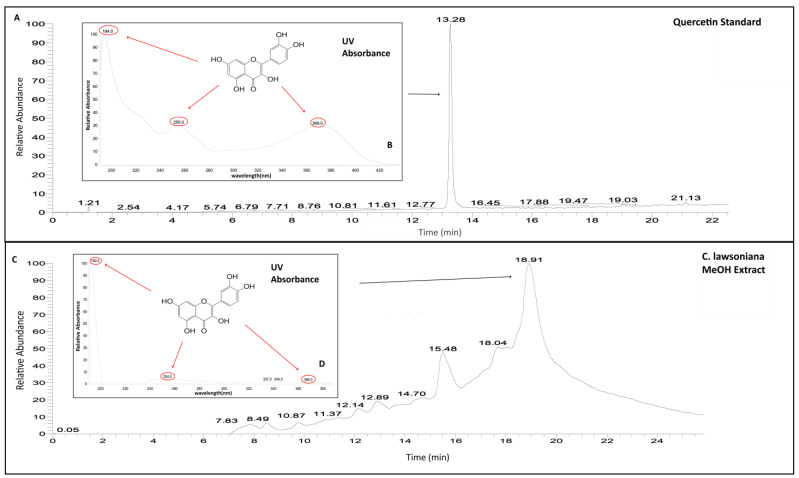
Phytochemical identification of quercetin from the MeOH extract of *C. lawsoniana.* Chromatograms for the standard (**A**) and MeOH extract of *C. lawsoniana* (**C**). Insets (**B**,**D**) illustrate the UV absorbance of the quercetin standard (**B**) and *C. lawsoniana* MeOH extract, respectively. Analysis revealed that the methanolic extract contained 0.019 mg (with a standard deviation of ±1.5209 × 10^−6^) of quercetin per 1 mg of the crude methanol extract. Additionally, the UV absorbance of quercetin in the *C. lawsoniana* methanolic extract, with a maximum wavelength (λmax) of 254.0 and 368.0 [MeOH], closely resembled that of the standard quercetin, which had a λmax of 255.0 and 369.0 [MeOH].

The aerial parts of the *C. lawsoniana* methanolic extract contained 0.019 mg (SD: ±1.5209 × 10^−6^) (y = 61.587x − 58.663; R^2^ = 0.9727) of quercetin (QC) in 1 mg of the crude methanol extract of the plant. The absorbance of QC under UV light (254 nm) of the *C. lawsoniana* methanolic extract (λmax 254.0; 368.0 [MeOH]) was similar to the absorbance of standard QC (λmax 255.0; 369.0 [MeOH]) (Figure 6). QC and derivate compounds have been reported in the *Chamaecyparis* genus. *C. lawsoniana* has also been reported to contain biflavones such as amentoflavone, bilobetin, isoginkgetin, and hinokiflavone [23] as well as 2,4‴-monomethylamentoflavone, 7,4′dimethylamentoflavone, 6,7″,4‴dimethylamentoflavone, and 7, trimethylamentoflavone [24]. Particularly, QC has been reported in *Chamaecyparis obtuse* [23,25], where QC derivates such as quercetin-3-O-α-rhamnopyranoside, myricetin-3-O-α-rhamnopyranoside [25], and quercitrin [26] have also been characterized.

### 2.7. Quercetin Inhibits the Contraction of the Airway Smooth Muscle 

**Figure 7:**
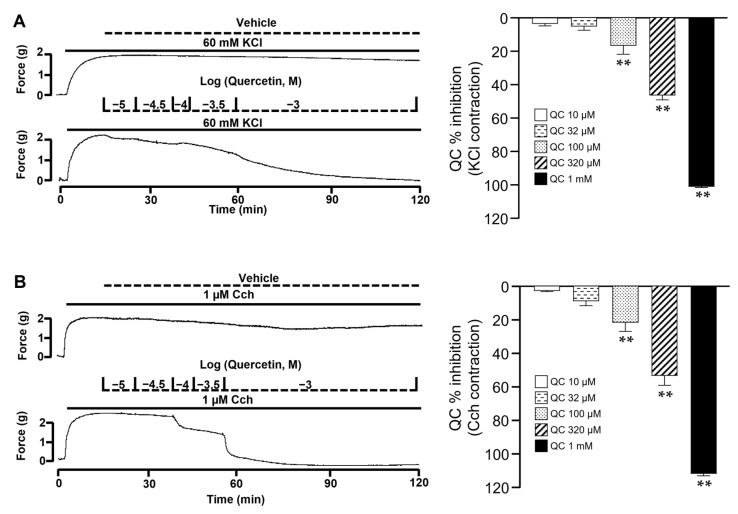
The contractions induced by either KCl or Cch in airway smooth muscle were relaxed by quercetin. (**A**,**B**) Quercetin, in a concentration-dependent manner, inhibited the contraction induced by KCl or Cch, respectively, in tracheal rings (*n* = 5). Bars represent mean ± SEM, ** *p* < 0.01.

QC induced the relaxation of the ASM precontracted with either KCl or Cch in a concentration-dependent manner (Figure 7). In the contraction with KCl (60 mM), the maximum inhibition was 101.13 ± 0.63% and the IC_50_ was 222.11 ± 8.96 µM (*n* = 5). The vehicle did not modify the contraction of the ASM. Additionally, in the Cch contraction, the maximum inhibition induced by QC was 112.03 ± 1.63%, and the IC_50_ was 204.01 ± 24.34 µM (*n* = 5).

### 2.8. Quercetin Blocks the Carbachol-Induced Ca^2+^ Plateau in Tracheal Myocytes

**Figure 8:**
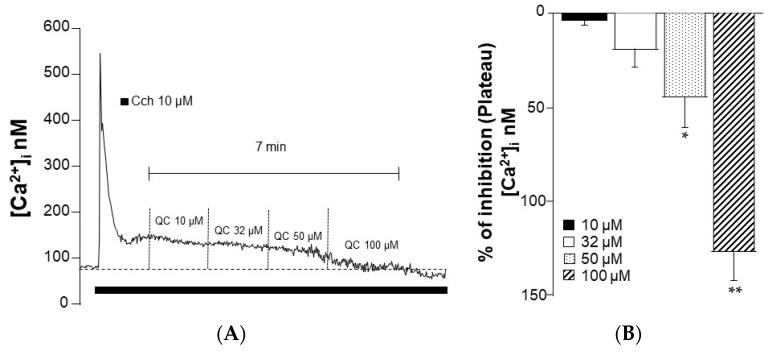
Quercetin blocks the carbachol-induced Ca^2+^ plateau. (**A**) In tracheal myocytes, carbachol (Cch) stimulation induced an intracellular Ca^2+^ peak followed by a plateau. The cumulative curve of quercetin (QC) diminished the Cch-induced plateau. (**B**) Bar graph illustrating statistical significance at 50 and 100 µM QC (*n* = 5). Bars represent mean ± SEM, * *p* < 0.05, ** *p* < 0.01.

Single tracheal myocytes stimulated with Cch (10 µM) produced a transient Ca^2+^ peak followed by a plateau (Figure 8A). Cumulative addition of QC concentrations lowered the Cch-induced Ca^2+^ plateau (*n* = 5), an inhibition that reached significance at 50 and 100 µM (Figure 8B).

## 3. Discussion

Our results indicate that *C. lawsoniana* inhibits the contractions induced by KCl, TEA, HIS, and Cch in ASM. The high K^+^ and TEA-generated contraction is mediated in its totality by L-VDCC; however, the response to HIS has an important L-VDCC component with a low IP_3_ participation [27,28]. On the other hand, the contractions induced by Cch are partially mediated by L-VDCC and SOCC [19,20,21,22,29]. The *C. lawsoniana* methanolic extract inhibited these channels and thus almost inhibited the contraction of ASM induced by KCl, TEA, and HIS, while it partially attenuated the contraction of ASM induced by Cch. 

We identified the pathways involved in the *C. lawsoniana*-induced inhibition of KCl, TEA, and HIS precontraction of ASM. In this regard, the addition of KCl to tracheal rings depolarizes smooth muscle, promoting L-VDCC opening, allowing Ca^2+^ entry to the cytoplasm, and inducing contraction, therefore remaining the preferred pharmacological tool to explore L-VDCC-related phenomena. We corroborated that the relaxation induced by *C. lawsoniana* was indeed through the inhibition of L-VDCC. In patch-clamp experiments, the tracheal myocytes were exposed to 18.75, 37.5, or 75 µg/mL *C. lawsoniana*, inducing a concentration-response reduction in the current. This current corresponded to L-VDCC activity since the addition of 1 µM nifedipine completely blocked the response. Additionally, the blockage of K^+^ channels with TEA also induced contraction because of membrane depolarization and L-VDCC opening. This last experimental maneuver allowed us to rule out the possible activation of K^+^ channels by the *C. lawsoniana* methanolic extract, as tracheal preparations were relaxed by *C. lawsoniana*, even when TEA blocked the K^+^ channels. Furthermore, through patch clamp experiments with tracheal myocytes, we corroborated that K^+^ currents were not altered by the *C. lawsoniana* methanolic extract at 150 µg/mL (Figure 4B). Studies carried out in our lab point out that in ASM, HIS-induced contractions have an important L-VDCC component with a low IP_3_ participation [27,28]; a fact that thoroughly explains *C. lawsoniana*-induced relaxations of the tracheal HIS-induced contraction described herein.

In the case of the result observed in the Cch-contraction, *C. lawsoniana* induced a partial inhibition (Figure 3). This response could be attributed to the differing mechanisms involved in the generation of the contraction by Cch, where SOCC and IP_3_ are known to participate in addition to L-VDCC [15,19,20], as demonstrated by the partial relaxation induced by the addition of nifedipine and 2-APB [19,20]. The individual inhibition of L-VDCC or SOCC in the Cch contraction does not correspond to the complete abolition of the response, but the consecutive addition of nifedipine and 2-APB does [19]. SOCC are non-selective cation channels (NSCC) that allow for the influx of Na^+^ and Ca^2+^ ions in ASM [29,30]. This large channel group is composed of ORAI1 and STIM1 proteins [31,32] as well as other channels (TRPC1, TRPC2, TRPC3, TRPC4, TRPC5, TRPC6, and TRPVs) in the ASM [33,34,35,36,37]. These channels participate in the depolarization of the plasma membrane; therefore, the inhibition of these channels could induce hyperpolarization of the cell, as has been previously described [30,36]. Since these channels were inhibited by *C. lawsoniana*, we concluded that they mediate the *C. lawsoniana*-induced relaxation in ASM. 

In this study, our aim was to investigate whether *C. lawsoniana* inhibits these channels, subsequently inducing relaxation. Our findings confirmed that *C. lawsoniana* effectively inhibited L-VDCC, leading to the observed relaxation, as detailed earlier. In addition, we corroborated in tracheal myocytes that *C. lawsoniana* could block in its totality the intracellular Ca^2+^ plateau induced by Cch, as observed in the Ca^2+^ measurements via microfluorometry (Figure 5). This plateau is known to be sustained by the influx of Ca^2+^ through the L-VDCC and SOCC [19]. In this context, the results suggest that *C. lawsoniana* can inhibit the L-VDCC and SOCC channels and thus block Cch-induced contraction. 

In addition, the *C. lawsoniana* methanolic extract was found to contain QC. Moreover, we demonstrated that QC inhibits the ASM contraction induced by KCl or Cch (Figure 7). Similarly to the *C. lawsoniana* extract, QC blocked the Cch-induced Ca^2+^ plateau (Figure 8), suggesting that the inhibitory mechanisms correspond to the effects observed for the *C. lawsoniana* extract. Researchers are considering QC to be one of the principal metabolites of the plant [38]. QC, a known key metabolite within plants, is a polyphenolic flavonoid termed 3,31,41,5,7-pentahydroxyflavone. This molecule occurs naturally in various fruits and vegetables such as onions, capers, apples, berries, tea, tomatoes, grapes, brassica vegetables, and shallots as well as in a multitude of nuts, seeds, barks, flowers, and leaves [38,39]. In this sense, QC is found in the ethyl acetate fraction of *Polygonum aviculare* L. (EAF), and both EAF and QC reduce mouse bronchial and tracheal and human bronchial ASM contraction induced with high K^+^ and Cch. The contraction by high K^+^ is completely dependent on the L-VDCC, and both L-VDCC and SOCC are implicated in the Cch-response. To elucidate the effect of EAF and QC on these individual mechanisms, they were evaluated separately in electrophysiological studies. EAF and QC inhibited the L-VDCC-mediated currents, and acetylcholine-induced the SOCC-mediated currents [40]. Similarly, in another study, QC caused the relaxation of human ASM precontracted with acetylcholine (ACh), HIS, KCl, or CaCl_2_ in a concentration-dependent manner. Additionally, QC potentiated the relaxation induced by isoprenaline and sodium nitroprusside on ACh-contracted human bronchial preparations [41]. Townsend et al. found that QC effectively inhibited the contraction induced by ACh in mice tracheal rings in a concentration-dependent manner [42]. QC potentiates the relaxation induced by isoproterenol in tracheal rings precontracted with ACh. Likewise, in an in vivo model, nebulization with quercetin significantly attenuated the methacholine challenge response. These relaxing effects were attributed to the inhibition of phosphodiesterase 4D (PDE4D) and phospholipase C β (PLCβ), since QC was shown to inhibit their activity [42]. To rule out other possible participating mechanisms, subsequent contraction studies were conducted in the presence of iberiotoxin (large-conductance Ca^2+^-activated K^+^ channel blocker), indomethacin (endogenous prostaglandin inhibitor), and L-NAME (an inhibitor of nitric oxide synthase). None of these drugs modified the effect of QC, suggesting that these mechanisms do not participate in the effect induced by it. Additionally, QC attenuates the intracellular Ca^2+^ response to G protein-coupled receptor agonists (histamine and bradykinin) [42]. 

Similarly, Hake et al. found that one of the main compounds of the *Drosera rotundifolia* L. extract was QC. The dry *Drosera* extract (DE) and an aqueous fraction (DFA) were used to examine its effects on ACh-induced contraction on mouse tracheal slices. Both DE and DFA reduced the maximum contraction induced by ACh. Likewise, isolated QC exerted a concentration-dependent relaxation of the ACh-induced contraction. This effect was attributed to the inhibitory activity of QC over PDE1A and PDE4D, with a comparable inhibition of PDE4D to rolipram (a PDE inhibitor) [43]. Standard QC could also inhibit ASM contraction and currents mediated by L-VDCC and ACh-activated NSCC [43]. 

Even though the essential oil components of *C. lawsoniana* have been extensively studied, a notable gap in research concerning the aerial parts remains. Exploring this aspect is crucial for a comprehensive understanding of the plant’s therapeutic potential. However, it is essential to acknowledge that our current study does not delve into the clinical implications or pharmacokinetics of the extract. Such investigations lie beyond the scope of our present research.

In this sense, our results suggest that *C. lawsoniana*, primarily through its active metabolite QC, can inhibit ASM contraction by inhibiting the L-VDCC and SOCC. Finally, the acute inhibition by *C. lawsoniana* of the L-VDCC and SOCC is reversible, corroborated by the Cch experiments in tracheal smooth muscle, where no alteration was noticed after tissue incubation with *C. lawsoniana*. Therefore, *C. lawsoniana* might possess the potential to counteract the dysfunctional Ca^2+^ mechanisms observed in respiratory pathological processes such as asthma, so further research regarding other *C. lawsoniana* chemical components is warranted.

## 4. Materials and Methods

### 4.1. Plant Material

*Chamaecyparis lawsoniana* (World Flora Online, https://www.worldfloraonline.org, accessed on 1 January 2024 ID: wfo-0000599456), Lawson cypress, Port Orford cedar, or ginger pine leaves were collected in the Mexican state of Puebla, county of Chignahuapan, in the town of Toltepam: Lat. 19.83710 N; Long. −98.034370 W. For its botanical identification, biological material was deposited at the Mexican National Herbarium at the Institute of Biology, UNAM (Voucher 1453965).

### 4.2. Methanolic Extraction Procedure 

The leaves of *C. lawsoniana* were dried at room temperature under shade. Then, the plants (363 g) were mechanically pulverized. Later, the powder was extracted by successive maceration 1:10 (*w*:*w*) with a CHCl_3_/MeOH mixture of solvents. At least five consecutive macerations were performed at 25 °C. Under reduced pressure, the methanolic extract underwent filtration and concentration, and residual solvent was removed using a rotary evaporator (Heildoph, Schwabach, Germany) at reduced pressure. The extract yield was determined by calculating the ratio of the obtained solids to the mass of the plant material employed in the extraction process. A yield of 13.93 g (3.83%) of crude MeOH extract was obtained. The methanolic extract was used to determine chemical and biological activity. Solutions used in this study were prepared by dissolving this extracted material in methanol to a final concentration of 5 mg/mL. This procedure is commonly used to rapidly obtain polar secondary metabolites in plants [44,45,46,47]. 

### 4.3. Quercetin Identification from C. lawsoniana MeOH Extract

The phytochemical analysis of the *C. lawsoniana* methanolic extract was made according to the method described by Estrella-Parra et al. [48]. An HPLC-DAD (Thermo Dionex Ultimate 3000, Thermo Fisher Scientific, Waltham, MA, USA) was used. Chromeleon and Xcalibur software were employed (Thermo Scientific Xcalibur V. 4.1.5.0). The method was as follows: a LiChrosorb column (250 mm × 4 mm, 10 μm; Hibar RT 250-4, Lot. L 347620) was used. The samples underwent analysis using a gradient comprising 0.1% formic acid in water (*v*/*v*) (A), 0.1% formic acid in acetonitrile (*v*/*v*) (B), and 0.1% formic acid in methanol (*v*/*v*) (C). The initial composition was 95% A, 2% B, and 3% C, transitioning to 54% A, 23% B, and 23% C after 15 min, and concluding with 95% A, 2% B, and 3% C at the 28-min mark. The flow rate was maintained at 0.6 mL/min. The quantification of the QC dehydrate was carried out using six different concentrations (40, 30, 20, 10, 5, 1 µg/mL), using the equation of the line (y = mx + b) to quantify the amount of QC in the methanolic extract. The *C. lawsoniana* methanolic extract injected was 20 µg (three different experiments).

### 4.4. Animals

Male Hartley guinea pigs (Cavia porcellus, 400–600 g) were bred in conventional conditions in our institutional animal facilities (filtered, conditioned air, 21 ± 1 °C, 50–70% humidity, sterilized bed) and fed with commercial pellets and sterilized water. The protocol was authorized by the Scientific and Bioethics Committees of the Facultad de Medicina, UNAM FM/DI/021/2018. The experiments were conducted in accordance with the published guidelines for the care and use of animals approved by the American Physiological Society (https://www.physiology.org/career/policy-advocacy/policy-statements/care-and-use-of-vertebrate-animals-in-research?SSO=Y, 2014, accessed on 21 November 2023) and the National Institutes of Health Guide for the Care and Use of Laboratory Animals [49]; the Mexican National Protection Laws on Animal Protection and the General Health Law Related to Health Research (NOM-062-Z00-1999) were also taken into consideration.

### 4.5. Organ Bath Studies

Animals were euthanized using pentobarbital sodium (35 mg/kg, i.p.) and subsequently exsanguinated. The trachea was carefully dissected and freed of connective tissue, yielding eight rings. These rings were suspended in a 5 mL organ bath containing Krebs solution (in mM): 2 CaCl_2_, 118 NaCl, 1.2 MgSO_4_, 1.2 KH_2_PO_4_, 25 NaHCO_3_, 4.6 KCl, and 11 glucose. The preparations were maintained at 37 °C and exposed to a 5% CO_2_ and oxygen mixture at pH 7.4. The tracheal segments were affixed to an isometric force transducer (model FT03; Grass Instruments, West Warwick, RI, USA) linked to a signal conditioning system (CyberAmp 380, Axon Instruments, Foster City, CA, USA) and an analog–digital interface (Digidata 1440A, Axon). Recordings were stored in a microcomputer and analyzed using data acquisition and analysis software (AxoScope, version 10.2, Axon). Prior to testing, all preparations were equilibrated for 30 min under a resting tension of 1 g. 

Experimental tissues were stimulated three times with KCl (60 mM). To evaluate the relaxing effect of *C. lawsoniana*, tracheal rings were precontracted with 60 mM KCl, 10 mM tetraethylammonium (TEA), 10 μM histamine (HIS), or 1 µM carbachol (Cch). In the case of the Cch experiments, once the maximal contraction response was reached, 30 µM methoxyverapamil (D-600), a blocker of the L-VDCC, was added before the addition of a single final concentration of 9.37, 18.75, 37.5, 75, 150, or 250 µg/mL of the methanolic extract of *C. lawsoniana* to evaluate its effects on store-operated Ca^2+^ channels (SOCC). To demonstrate that the effects of *C. lawsoniana* were acute and reversible, after an initial incubation with the extract, all tracheal preparations received a KCl stimulus after washout. This last response was similar to the third KCl response. The addition of the highest methanol volume employed (50 µL, 0.1%) to the tissue preparations in 5 mL organ bath chambers did not modify the tracheal contractions to KCl or TEA. To evaluate the relaxation induced by quercetin (QC), a cumulative concentration–response curve to QC (10, 32, 100, 320, and 1000 µM) added to the tracheal rings precontracted with 60 mM KCl or 1 µM carbachol (Cch) was undertaken.

In the summary graphs of the relaxation, the maximum contraction point was taken as 0%, and 100% represents the basal tone.

### 4.6. Patch Clamp Recordings

Myocytes were obtained from the trachea of guinea pigs using the following procedure: guinea pig tracheal smooth muscle without fat and connective tissue was placed in 5 mL Hanks solution supplemented with 2 mg L-cysteine and 0.05 U/mL papain and incubated at 37 °C for 10 min. The tissue was then rinsed with Leibovitz solution without phenol red from GIBCO (contains glutamine, sodium pyruvate, and galactose) to remove excess enzymes, and transferred to Hanks solution from GIBCO containing 1 mg/mL collagenase type I and 4 mg/mL dispase II (neutral protease) before being digested at 37 °C for about 20 min. The smooth muscle was then gently dispersed until individual cells detached, and the enzymatic activity was stopped by adding Leibovitz solution. The cells were centrifuged for 5 min at 800 rpm at 20 °C, and the supernatant was discarded. This centrifugation step was repeated once. To culture myocytes, the cell pellet was suspended in minimal essential medium (MEM) from GIBCO, supplemented with fetal bovine serum (10%), 2 mM L-glutamine, 10 U/mL penicillin, 10 μg/mL streptomycin, and 15 mM glucose. The cells were then plated on round coverslips coated with sterile rat tail collagen and cultured at 37 °C with 5% CO_2_ in oxygen for 24 to 48 h.

We allowed the ASM cells to settle at the bottom of a 0.7-mm coverslip in a perfusion chamber to facilitate the experiments. The chamber was then perfused by gravity at a rate of approximately 1.5–2.0 mL/min with an external solution containing Ba^2+^ (inward charge carrier) as a substitute for Ca^2+^ to measure the Ca^2+^ currents. This solution consisted of (in mM): NaCl (136), CsCl (6), BaCl_2_ (5), glucose (11), HEPES (10), and niflumic acid (0.1), with the pH adjusted to 7.4 with CsOH. A different external solution was used to record the K^+^ currents, consisting of (in mM): NaCl (130), KCl (5), CaCl_2_ (1), HEPES (10), glucose (10), MgCl_2_ (0.5), NaHCO_3_ (3), KH_2_PO_4_ (1.2), and niflumic acid (0.1), with the pH adjusted to 7.4 with NaOH. All experiments were carried out at room temperature (~21 °C). Whole-cell recordings of the Ba^2+^ or K^+^ currents were performed using the standard whole-cell configuration and an Axopatch 200A amplifier (Axon Instruments). Patch pipettes were made of 1B200F-6 glass using a horizontal micropipette puller (P-87, Sutter Instruments Co., Novato, CA, USA). They had 2 to 4 MΩ resistances when filled with specific internal solutions for measuring Ba^2+^ or K^+^ currents. The internal solution for Ba^2+^ currents contained (in mM): CsCl (130), MgCl_2_ (2), HEPES (10), EGTA (10), ATP sodium salt (3.6), and GTP sodium salt (1.9), with the pH adjusted to 7.3 with CsOH. The internal K^+^ solution consisted of (in mM): potassium gluconate (140), NaCl (5), HEPES (5), EGTA (1), ATP sodium (5), GTP sodium (0.1), and leupeptin (0.1), with the pH adjusted to 7.3 with KOH. Whole-cell currents were filtered at 1–5 KHz, digitized with a digitizer (Digidata 1440A, Axon Instruments) at 10 KHz, and then stored on a computer for subsequent analysis with the software pClamp, version 10.2.

To record the Ba^2+^ and K^+^ currents, a series of depolarizing pulses from −60 to +50 mV in 10 mV steps were applied to tracheal myocytes at a frequency of 1 Hz from a holding potential of −60 mV for 500 ms. Following the control depolarizing-pulses protocol, different concentrations of the methanolic extract of *C. lawsoniana* (18.75, 37.5, and 75 µg/mL final concentration in the perfusion solution, *n* = 5 each) were administered, and the same experimental procedure was repeated. The L-type Ca^2+^ currents were then characterized with nifedipine (1 µM) at the end of the experiments. In further experiments, outward K^+^ currents were studied under the same depolarization protocol. The effect of the *C. lawsoniana* extract on these currents was investigated with a final concentration of 150 µg/mL (*n* = 3). Current changes were evaluated based on the maximum current peak for each voltage tested. The addition of the highest amount of methanol used (30 µL, 0.03% *v*:*v*) did not alter these inward currents).

### 4.7. Intracellular Ca^2+^ Measurements in Tracheal Myocytes

Guinea pig tracheal myocytes, isolated as previously outlined, were incubated in a low Ca^2+^ (0.1 mM) solution at approximately 21 °C for 1 h and subsequently loaded with 2.5 µM fura 2-AM. Following this, the cells were transferred to a heated perfusion chamber with a glass cover at the bottom, affixed to an inverted microscope (Diaphot 200, Nikon, Tokyo, Japan). The myocytes adhered to the glass cover were perfused at a rate of 2–2.5 mL/min with Krebs solution at 37 °C, continuously exposed to a 5% CO_2_ and oxygen mixture at pH 7.4.

Excitation light pulses of 340/380 nm were applied to these myocytes, and emission light at 510 nm was collected using a microphotometer from Photon Technology International, model D-104 (PTI, Princeton, NJ, USA). The Grynkiewicz formula, with a supposed Kd for fura 2-AM of 386 nM [50], was employed to calculate intracellular Ca^2+^ concentration ([Ca^2+^]i). Fluorescence was recorded at intervals of 0.5 s, and the mean 340/380 fluorescence ratios Rmax and Rmin were determined to be 8.99 and 0.352, respectively. Data were stored in a computer for subsequent analysis using specialized software (Felix, version 1.21, PTI).

Myocytes were stimulated with 10 µM Cch, and during the Ca^2+^ plateau, cumulative concentrations of the methanolic extract of *C. lawsoniana* (37.5, 75, and 150 µg/mL) were perfused. To determine whether the effects of *C. lawsoniana* were acute and reversible, myocytes were washed with Krebs solution for 20 min and received a second 10 µM Cch stimulation. This second response was similar to the first Cch response before the addition of *C. lawsoniana*, corroborating acute inhibition and reversibility. In another set of experiments, during the Ca^2+^ plateau induced by Cch, D-600 (30 µM, an L-VDCC blocker), 2-aminoethyl diphenylborinate (2-APB, 100 µM, a SOCC blocker), and QC (10–100 µM) were added. 

### 4.8. Drugs and Reagents

Carbamylcholine chloride (carbachol), histamine dihydrochloride, tetraethylammonium, methoxyverapamil, nifedipine, quercetin and methanol were obtained from Sigma Chemical Co. (St. Louis, MO, USA). 2-Aminoethyl diphenylborinate was purchased from Tocris Bioscience (Ellisville, MO, USA). Collagenase type I and papain were purchased from Worthington Biochemical Co. (Lakewood, NJ, USA). Dispase II was obtained from Roche (Indianapolis, IN, USA). 

### 4.9. Data Analysis

Comparison among groups was carried out through a one-way analysis of variance followed by Dunnett’s test or paired Student t-test. Statistical significance was set at *p* < 0.05 bimarginally. Data in the text and figures correspond to the mean ± SEM.

## 5. Conclusions

*C. lawsoniana* relaxes the contracted ASM by blocking the Ca^2+^ influx through SOCC and L-VDCC. QC is the main active compound of *C. lawsoniana* responsible for these inhibiting mechanisms, and our results suggest that it could potentially be a new bronchodilator agent.

## Data Availability

Data available on request from the authors.
